# Case report: Methemoglobinemia caused by nitrobenzene poisoning

**DOI:** 10.3389/fmed.2023.1096644

**Published:** 2023-02-21

**Authors:** Liwen Zhao, Tianzi Jian, Longke Shi, Yaqian Li, Zixin Wen, Lanlan Guo, Qilu Li, Xiangdong Jian

**Affiliations:** ^1^Department of Occupational and Environmental Health, School of Public Health, Cheeloo College of Medicine, Shandong University, Jinan, Shandong, China; ^2^Department of Poisoning and Occupational Diseases, Emergency Medicine, Qilu Hospital, Cheeloo College of Medicine, Shandong University, Jinan, Shandong, China; ^3^Department of Hematology, Qilu Hospital, Cheeloo College of Medicine, Shandong University, Jinan, Shandong, China; ^4^School of Nursing and Rehabilitation, Cheeloo College of Medicine, Shandong University, Jinan, Shandong China; ^5^Nursing Theory and Practice Innovation Research Center, Shandong University, Jinan, Shandong, China; ^6^Department of Nursing, Qilu Hospital of Shandong University, Jinan, Shandong China; ^7^The Hospital of Shandong University, Jinan, Shandong, China

**Keywords:** nitrobenzene, methemoglobinemia, hemolytic anemia, toxic encephalopathy, cyanosis

## Abstract

Nitrobenzene poisoning is uncommon, with most cases occurring in the dye, paint, and other chemical industries. Nitrobenzene enters the body mainly through the skin, respiratory tract, and oral cavity. Nitrobenzene poisoning symptoms include hypermethemoglobinemia, hemolytic anemia, liver and kidney dysfunction, cardiogenic pulmonary edema, and toxic encephalopathy, which endanger people’s lives. Therefore, we present a case of nitrobenzene poisoning caused by skin absorption, focusing on its clinical characteristics and treatment outcomes. A 58 years-old man presented to our department with confusion and cyanosis. He has a history of hypertension and cerebral infarction. The patient was diagnosed with moderate occupational acute benzene poisoning with nitro compounds. Symptomatic support, methylene blue, and other antioxidant treatments were commenced after diagnosis. After treatment, the patient’s condition gradually improved, and he was discharged.

## 1. Introduction

Nitrobenzene (C_6_H_5_NO_2_), also known as dense spot oil and bitter almond oil, is a colorless or slightly yellow oily liquid with a bitter almond taste. It is water-insoluble but soluble in ethanol, ether, benzene, and oil (1). Nitrobenzene is often used in the production of aniline, explosives, dyes, spices, coatings, medicine, and pesticides, and may be exposed to the environment during the use, loading, unloading, and handling of raw materials. It is mainly absorbed through the skin during production, but the vapor can also be absorbed through the respiratory tract. The risk of inhalation poisoning increases when the temperature rises (2, 3). Patients with acute poisoning may exhibit obvious cyanosis, palpitations, chest tightness, dyspnea, and other hypoxia symptoms. Furthermore, high fever, sweating, convulsions, and coma may occur in severe cases. In mild disease without anemia, severe poisoning can cause hemolytic anemia, jaundice, and liver damage after a few days. Brain lesions may occur in a few patients. Therefore, this study aimed to describe a case of nitrobenzene poisoning caused by skin absorption, focusing on its clinical characteristics and treatment outcomes.

## 2. Case presentation

**FIGURE 1:**
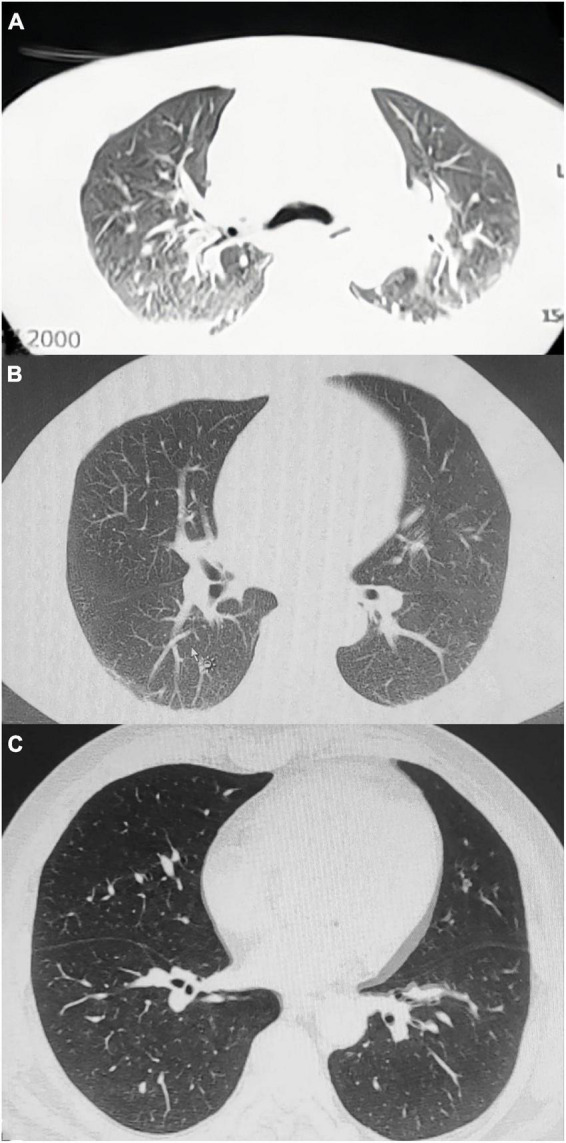
Computed tomography changes in the patient’s lungs. **(A)** Increased and disordered bronchovascular bundles in both lungs, blurred and poorly defined boundaries, multiple patchy high-density shadows, mainly in the lower lobe, and curved fluid density shadows in the bilateral thorax. **(B)** Increased texture of both lungs, patchy high-density shadows on the dorsal side of both lungs, multiple solid microscopic nodules in both lungs, and a small fluid density in the bilateral thorax. **(C)** Multiple micronodules are observed in both lungs, and a few fibrous foci are observed in the left lung.

A 58 years-old man presented to our department with confusion and cyanosis for approximately 8 h. His weight is 80 kg. The patient had been working in the technical production of 4-dinitrodiphenyl ether at a local insulation material company for 11 years. The main operation processes included condensation, reduced pressure distillation, water solution, extraction, and filtration (Supplementary material). The patient was exposed to substances, including nitrobenzene, p-nitrochlorinated benzene, sodium p-nitrophenol, and potassium chloride. A similar case was reported in the unit 1 week ago. On August 18, 2022, after returning home from work, the patient felt dizzy at 20:10 p.m. and then lost consciousness. He was unresponsive to the callout and had circumoral cyanosis. He was rushed to the nearest hospital at 21:10 p.m. The patient’s temperature then was 36.7°C, pulse rate was 88 beats/min, respiration rate was 23 beats/min, blood pressure was 129/94 mmHg, and oxygen saturation was 86%. The patient’s arterial blood gas analysis revealed 70% methemoglobin, and computed tomography (CT) revealed bilateral pleural effusion and pneumonia (Figure 1). The patient was immediately treated with methylene blue, vitamin C, and methylprednisolone (the specific dose is unknown). The patient’s cyanosis improved after treatment, and he was transferred to our hospital at 16:49 p.m. on August 19, 2022.

**FIGURE 2:**
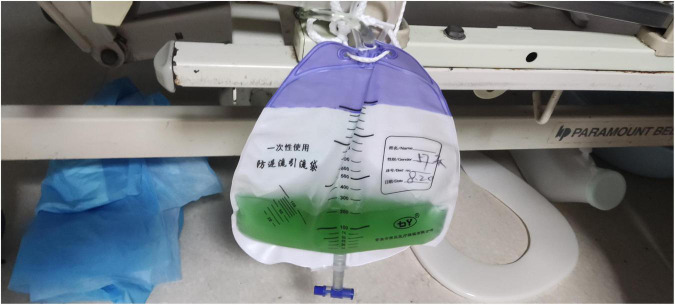
The urine color after administering methylene blue.

**FIGURE 3:**
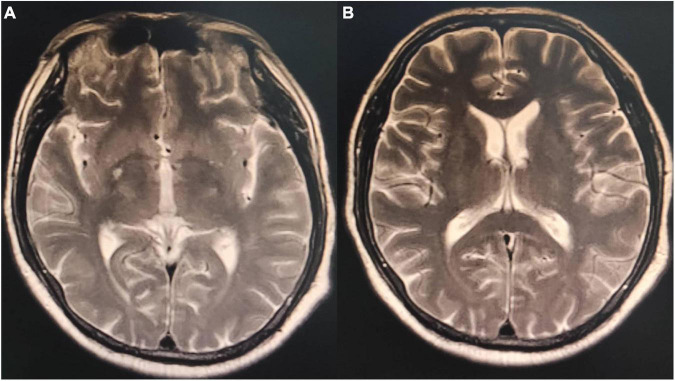
Magnetic resonance imaging T2 phase of the patient’s brain. **(A)** Small patches of the long T2 signal shadow in the right basal ganglia with hyperintensity in T2 fluid-attenuated inversion recovery (FLAIR) and a few patchy long T2 signals in the white matter of bilateral cerebral hemispheres with hyperintensity in T2 FLAIR. Acute lacunar infarction in the right basal ganglia might be large, and there were few ischemic degeneration foci in the white matter of the bilateral cerebral hemispheres. **(B)** Small patches of long T2 signal shadow were observed in the right basal ganglia, with slight hyperintensity in T2 FLAIR. A few patchy long T2 signals were observed in the white matter of the bilateral cerebral hemispheres, with hyperintensity in T2 FLAIR. A few ischemic degeneration foci in the white matter of bilateral cerebral hemispheres and ischemic infarction foci in the right basal ganglia were considered.

On admission, the patient’s temperature was 36.5°C, pulse rate was 83 beats/min, respiration rate was 21 beats/min, blood pressure was 85/65 mmHg, and oxygen saturation was 85%. He had been taking oral antihypertensive drugs for more than 10 years. However, the specific medications and control status were unknown. He has a 1 year history of cerebral infarction, with no residual limb movement disorder. He has no congenital or genetic disorders. The patient was lethargic and uncooperative during the physical examination. There was no yellowing or breaking of the skin or mucous membranes in the whole body. Cyanosis was evident in the nails. The pupils on both sides were similar in size (large) and shape (round). Moreover, no neck weakness, or resistance was observed. There was equal chest expansion, but there were weakened breath sounds in both lungs, and a small number of wet rales was heard in the middle and lower lungs. The abdomen was soft and the bowel sounds were normal. The fixed indwelling catheter revealed blue-green urine (Figure 2). No pathological reflex was elicited. Arterial blood gas examination revealed the following: pH 7.43, pCO2 39 mmHg, pO2 91 mmHg, sO2 90.0%, HCO3^–^ 25.9 mmol/L, methemoglobin (reference value 0–3%) 11.1%, reduced hemoglobin (reference value 0–5%) 8.8%, and oxygenated hemoglobin (reference value 95–98%) 79.5%. Electrocardiogram revealed J-point depression and borderline first degree atrioventricular block (Supplementary material). Simultaneously, relevant auxiliary examinations improved (Table 1). According to the diagnostic criteria of the National Occupational Health Standards (4), the patient was diagnosed with moderate occupational acute benzene poisoning with nitro compounds. Hence, he was given dexamethasone sodium phosphate 20 mg injection followed by intravenous infusion q.d. and methylene blue 80 mg stat micropumped and maintained for 15 min. Symptomatic treatments, such as oxygen inhalation, fluid replenishment, hepatoprotective diuresis, awakening, and nutritional support were provided, and HA330 hemoperfusion therapy was administered after the vital signs had stabilized. Following treatment, the patient woke up on the same day and was able to answer questions freely. A brain CT scan revealed no abnormalities. On the 7th day of admission, he complained of dyspnea, and his oxygen saturation was maintained at 92% under oxygen inhalation. Laboratory tests revealed mild anemia and elevated bilirubin levels. Therefore, he was administered a polysaccharide iron complex capsule 0.15 g orally q.d. Magnetic resonance imaging (MRI) of the brain revealed a possible acute lacunar infarction in the right basal ganglia and a few ischemic degenerations in the white matter of both hemispheres (Figure 3). On the 11th day of admission, the patient’s breathlessness had not improved significantly, and arterial blood gas revealed 8.1% methemoglobin, pH 7.42, pCO2 45 mmHg, pO2 100 mmHg, sO2 97.7%, and HCO3^–^ 29.2 mmol/L. Subsequently, 20 mg of methylene blue was administered and maintained *via* micropump for 15 min b.i.d. Additionally, an intravenous infusion of 3.0 g of vitamin C q.d. was administered. After treatment, the patient’s condition gradually improved, and he was discharged from the hospital on September 4, 2022. The patient was instructed to do regular follow-up after discharge. However, due to the novel coronavirus epidemic, the patient did not return to our hospital. One month after discharge, our department made a telephone follow-up and the patient complained of weakness but had no episode of suffocation. Three months after discharge, the patient reported that he had no discomfort and had good quality of life.

**TABLE 1:** Dynamic changes in the patient’s laboratory examination.

Project	Reference range	Day 1	Day 3	Day 7	Day 14
WBC (10*9/L)	3.5–9.5	**12.18**	**14.92**	8.48	**10.2**
NEU% (%)	40–75	**91.1**	**77.6**	63.4	60
LYM% (%)	20–50	**6.1**	**16.4**	28.5	32.9
RBC (10*12/L)	4.3–5.8	**3.43**	**3.53**	**3.00**	**2.69**
HGB (g/L)	130–175	**107**	**106**	**94**	**89**
ALT (U/L)	9–50	27	16	31	29
AST (U/L)	15–40	41	16	19	21
TBIL (μmol/L)	5.0–21.0	**48**	**37.3**	**34.8**	**37.4**
DBIL (μmol/L)	<6.0	0	**10.0**	**10.3**	**9.8**
IBIL (μmol/L)	2.0–15.0	**43**	**27.3**	**24.5**	**27.6**
BUN (mmol/L)	2.3–7.8	6.7	7.4	6.3	7
Cr (μmol/L)	62–115	51	56	55	56
CK (U/L)	38–174	132	65	15	24
CK-MB (ng/mL)	0.3–4.0	–	1.6	0.4	0.4
CTNI (ng/L)	<17.5	–	**506.03**	–	4.28
LDH (U/L)	120–230	227	**326**	**245**	**324**
DD-i (μ g/mL)	<0.50	**0.92**	**1.81**	**5.73**	**4.94**

WBC, white blood cells; NEU%, neutrophil ratio; LYM%, lymphocyte ratio; RBC, red blood cells; HGB, hemoglobin; ALT, alanine aminotransferase; AST, aspartate aminotransferase; TBIL, total bilirubin; DBIL, direct bilirubin; IBIL, indirect bilirubin; BUN, blood urea nitrogen; Cr, serum creatinine; CK, creatine kinase; CK-MB, creatine kinase isoenzyme; CTNI, hypersensitive troponin I; LDH, lactate dehydrogenase; DD-i, D-dimer. Bold values are outside the normal reference range and are outliers.

## 3. Discussion

Nitrobenzene is an oxidizing nitrite compound (5) that can cause human poisoning when 35 mg/kg is absorbed. The skin absorbs nitrobenzene at 2 mg/(cm^2^⋅h) rate. Moreover, nitrobenzene vapor can be absorbed *via* the skin and respiratory tract simultaneously, with a total retention rate in the body reaching 80% (6). Methemoglobinemia caused by nitrobenzene poisoning is the main pathological basis of acute poisoning. The intermediate substance formed during its biological transformation in the body causes red blood cell rupture and hemolysis. After entering the human body, nitrobenzene is transformed to produce intermediate products, which can lead to the reduction of reduced glutathione. Reduced glutathione plays an important role in the survival of red blood cells, such as in the maintenance of the normal function of red blood cell membrane. When reduced glutathione is decreased, it is easy to cause hemolysis. The intermediates also directly act on the basophobia in globin molecules to promote denaturation. The denatured globin condenses into precipitates and forms inclusion bodies (Hearn corpuscles) in red blood cells. The red blood cells containing Hearn corpuscles are easily ruptured, which is another reason for hemolysis. The red blood cell count decreases rapidly within 3-4 days but gradually increases after 1-2 weeks with active treatment. Furthermore, nitrobenzene poisoning can cause liver and kidney dysfunction, which usually occurs 2–3 days after the poisoning. Other symptoms include cardiogenic pulmonary edema and multiple organ dysfunction (7). The neurological symptoms of nitrobenzene poisoning are apparent, with excitation symptoms such as dizziness, headache, lethargy, and coma occurring earlier. The initial symptoms of the patient were obvious, which included high fever in the summer, acute attack after several hours of skin exposure, loss of consciousness, coma, and lip cyanosis. The patient had a history of cerebral infarction, but no obvious neurological symptoms occurred during hospitalization. Moreover, no abnormalities were observed in the early brain CT. However, MRI revealed right basal ganglia and bilateral white matter lesions 7 days after admission, indicating toxic encephalopathy caused by nitrobenzene poisoning. Current neurologic symptoms (headache, confusion, and coma) after nitrobenzene poisoning are usually interpreted as methemoglobinemia complications from the resulting cerebral hypoxia. However, some studies suggested that CT and MRI reveal targeted brain lesions rather than hypoxic encephalopathy. Moreover, toxic encephalopathy occurs more in the cerebellum and brainstem, and most of the lesion sites are symmetrically distributed (8). However, other sites cannot be excluded.

Acute nitrobenzene poisoning may occur during work or after work following an incubation period of several hours. When methemoglobin levels reach 10-15%, cyanosis develops in the patient’s mucosa and skin. When methemoglobin levels exceed 30%, other neurological symptoms, including head heaviness, dizziness, headache, tinnitus, finger numbness, and general weakness occur. When methemoglobin levels increase to 60–70%, the patient may experience shock, arrhythmia, convulsions, and coma. After the timely rescue, consciousness can be restored within 24 h, and pulse and respiration will then gradually improve. However, dizziness, headache, and other symptoms can last for days. Methemoglobin lethal concentration ranges from 85 to 90% (9), and the mortality rate is high if untreated.

The patient had 70% methemoglobin initially, with cyanosis of the skin and mucosa. The patient was in a coma and critical condition. Immediately after the patient was transferred to our hospital, methylene blue was administered, the patient’s symptoms improved significantly, and the methemoglobin level reduced to 11.1%. The current recommended treatment for nitrobenzene poisoning is based on decontamination principles and symptomatic and supportive treatments. Methylene blue is the first choice of antidotes for acquired (toxic) methemoglobin, which serves as an electron acceptor after entering the body to reduce methemoglobin to hemoglobin (10), which can participate in oxygen transport. However, excessive administration of methylene blue may cause accumulation in the body, resulting in secondary methemoglobinemia. At the same time, G6PD level should be tested because methylene blue is not recommended for patients with G6PD deficiency. Within 3–6 h after injecting methylene blue into the human body, blue-green urine is observed (11). Gradually, the color becomes light green-yellow to yellow to pale yellow and then becomes light yellow after approximately 72–96 h. Nicotinamide adenine dinucleotide phosphate metabolizes methylene blue into leucomethylene blue, which is mainly excreted in the urine, turning the urine color blue-green (12). Some scholars believe that it may be related to biliverdin, an *in vivo* bilirubin oxidation product (13, 14).

Ascorbic acid, an antioxidant, can be used in patients with methemoglobin levels exceeding 30% (15). In recent studies (7), N-acetylcysteine has been shown to reduce hemoglobin; however, it has not been approved for methemoglobinemia treatment. Exchange transfusion therapy is suitable for severe cases (16). Currently, hyperbaric oxygen therapy is reserved for patients with methemoglobin levels at 50% or those who did not respond to standard therapy (17). However, even after aggressive treatment, chest tightness and dyspnea may continue for a long time.

In the present case, hemolysis occurred in the early stage of the condition, decreasing red blood cells and hemoglobin. However, this progressed to moderate anemia, and bilirubin significantly increased and did not decrease further. The patient received fluid rehydration, hemoperfusion, and intravenous infusion of methylene blue and vitamin C promptly, and his symptoms improved significantly with no pertinent changes in vital signs. However, dyspnea and suffocation persisted and did not improve after high-flow oxygen inhalation. Fortunately, the patient did not have cardiac and renal injuries and multiple organ dysfunction; and eventually, he recovered and was discharged.

This case suggests that nitrobenzene poisoning is relatively rare. However, it should be treated symptomatically after diagnosis with the early administration of methylene blue. If the regional hospital has limited resources, a timely administration of an antioxidant and blood perfusion should be performed. Hemoperfusion is mainly used to relieve toxic poisoning and is mainly used for chemicals with large molecular weight, high fat solubility, and high protein binding rate. It is usually the first choice of treatment for acute poisoning. It is mainly prepared into a neutral macroporous resin adsorbent by suspension polymerization. The affinity between the resin molecule, the adsorbed substance, and the rich, three-position mesh molecular sieve adsorbs the medium and large molecular toxins as well as the toxins binding with proteins in human body. Early treatment can facilitate the removal of toxins absorbed by the body as soon as possible. Simultaneously, secondary methemoglobinemia should be prevented, and brain lesions should be observed as they might suggest toxic encephalopathy. The company should make a good prevention and control plan, strengthen the supervision and management of the operation process, install ventilation equipment, train workers to be familiar with the production process, install nitrobenzene air alarm in key positions as necessary, and provide timely monitoring. Workers should raise their awareness of personal protection and carry out trainings on occupational health so as to reduce the recurrence of similar incidents.

The limitation of the study was that due to coronavirus disease 2019 restrictions, the concentration of nitrobenzene in the patient’s workplace and blood were not collected, and only video data was used as evidence.

## Data availability statement

The original contributions presented in this study are included in the article/Supplementary material, further inquiries can be directed to the corresponding authors.

## Ethics statement

The studies involving human participants were reviewed and approved by Qilu Hospital of Shandong University. The patients/participants provided their written informed consent to participate in this study. Written informed consent was obtained from the individual(s) and/or minor(s)’ legal guardian/next of kin for the publication of any potentially identifiable images or data included in this article.

## Author contributions

LZ and TJ wrote the first draft of the manuscript. XJ and QL performed the final checks. LS, ZW, and YL prepared the material, data collection, and performed the analysis. All authors contributed to the study conception and design, commented on previous versions of the manuscript, read, and approved the final manuscript.
