# Supplementary figures and images for: Case report: Methemoglobinemia caused by nitrobenzene poisoning
Source: Front Med (Lausanne). 2023 Feb 21;10:1096644. doi: 10.3389/fmed.2023.1096644 (PMC9988939; doi:10.3389/fmed.2023.1096644)

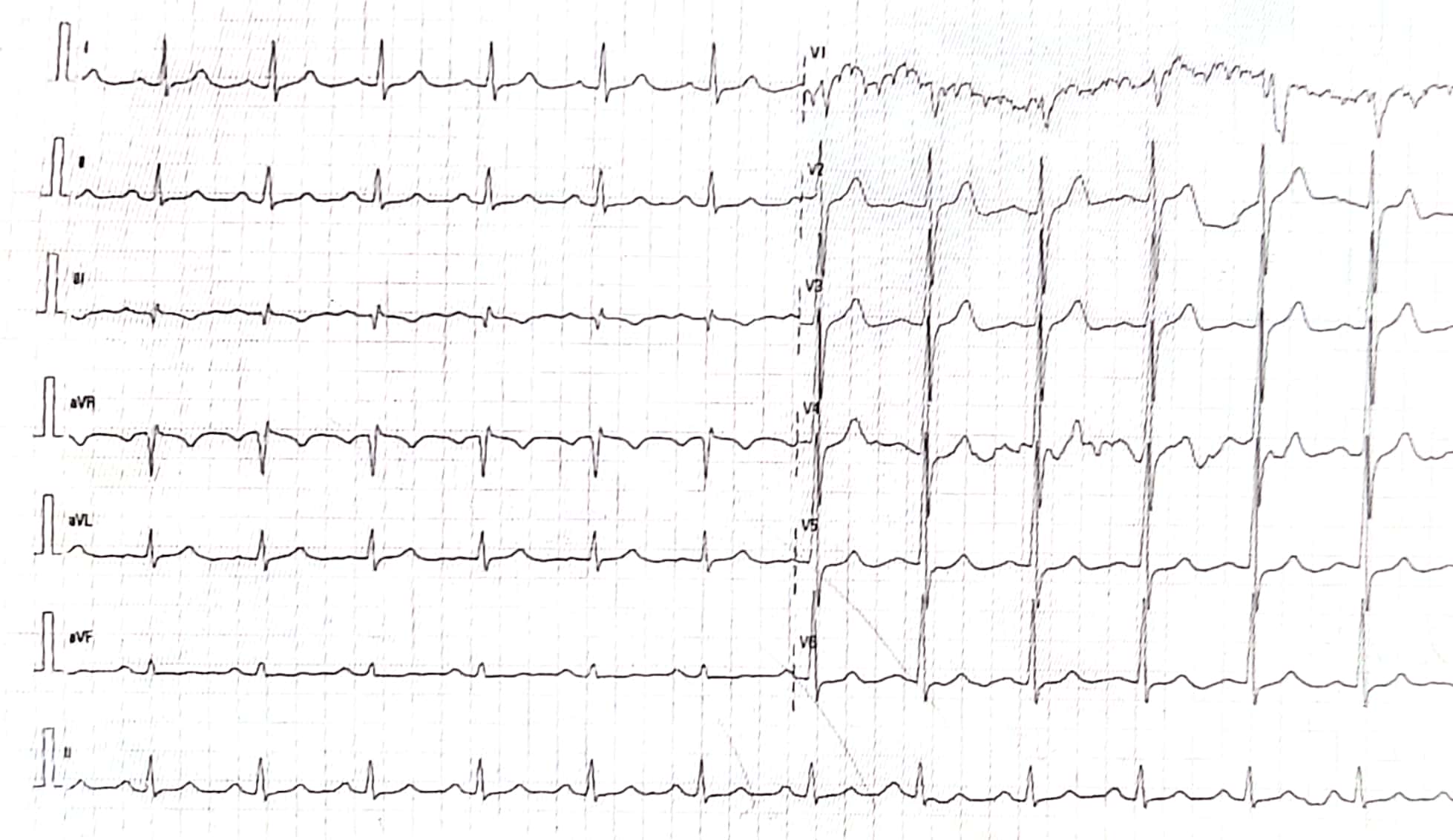

Supplement: Supplementary file 1 [file Image_1.JPEG]
